# Association of Mandatory Warning Signs for Cannabis Use During Pregnancy With Cannabis Use Beliefs and Behaviors

**DOI:** 10.1001/jamanetworkopen.2023.17138

**Published:** 2023-06-14

**Authors:** Sarah C. M. Roberts, Claudia Zaugg, M. Antonia Biggs

**Affiliations:** 1 Advancing New Standards in Reproductive Health, Department of Obstetrics, Gynecology and Reproductive Sciences, University of California, San Francisco, Oakland

## Abstract

**Importance:**

As states have legalized recreational cannabis use, some have enacted policies mandating point-of-sale warning signs with information on harms of using cannabis during pregnancy. While research has found such warning signs are associated with increased adverse birth outcomes, reasons why are unclear.

**Objective:**

To examine whether exposure to cannabis warning signs is associated with cannabis-related beliefs, stigma, and use.

**Design, Setting, and Participants:**

This cross-sectional study used data from a population-based online survey conducted from May to June 2022. Participants included pregnant and recently pregnant (within past 2 years) members of the national probability KnowledgePanel and nonprobability samples in all US states and Washington, the District of Columbia, where recreational cannabis is legal. Data were analyzed from July 2022 to April 2023.

**Exposure:**

Living in 1 of 5 states with a warning signs policy.

**Main Outcomes and Measures:**

Outcomes of interest were self-reported beliefs (linear) that cannabis use during pregnancy is not safe, should be punished, and is stigmatized and cannabis use during pregnancy (dichotomous). Regressions, accounting for survey weights and clustering by state, examined associations of warning signs with cannabis-related beliefs and use.

**Results:**

A total of 2063 pregnant or recently pregnant people (mean [SD] weighted age, 32 [6] years) completed the survey, and 585 participants (weighted, 17%) reported using cannabis during their pregnancy. Among people who used cannabis during their pregnancy, living in a warning signs state was associated with beliefs that cannabis use during pregnancy was safe (β = −0.33 [95% CI, −0.60 to −0.07]) and that people who used cannabis during pregnancy should not be punished (β = −0.40 [95% CI, −0.73 to −0.07]). Among people who did not use cannabis before or during pregnancy, living in a warning signs state was associated with beliefs that use was not safe (β = 0.34 [95% CI, 0.17 to 0.51]), that people should be punished for use (β = 0.35 [95% CI, 0.24 to 0.47]), and that use was stigmatized (β = 0.35 [95% CI, 0.07 to 0.63]). Warning signs policies were not associated with use (adjusted odds ratio, 1.11 [95% CI, 0.22 to 5.67]).

**Conclusions and Relevance:**

In this cross-sectional study of warning signs and cannabis-related use and beliefs, warning signs policies were not associated with reduced cannabis use during pregnancy or with people who used cannabis believing use during pregnancy was less safe but were associated with greater support for punishment and stigma among people who did not use cannabis.

## Key Points

**Question:**

Are policies mandating warning signs regarding the harms of cannabis use during pregnancy associated with beliefs and behaviors?

**Findings:**

This cross-sectional study used data from 2063 US-based survey respondents who were or had recently been pregnant and found that, among people who used cannabis during pregnancy, living in a state with a warning signs policy was significantly associated with believing use was safe and should not be punished; among those who did not use cannabis, warning signs policies were significantly associated with believing use was not safe and should be punished. There were no associations of living in a state with a mandatory warning sign policy with using cannabis during pregnancy.

**Meaning:**

These findings suggest that while warning signs policies were not associated with believing use was less safe among pregnant individuals who used cannabis during pregnancy, policies were associated with support for punishment among people who did not use cannabis.

## Teaser

This cross-sectional study examines associations of polices mandating warning signs on the harms of cannabis use during pregnancy with cannabis use and behaviors among pregnant or recently pregnant survey respondents.

## Introduction

As recreational cannabis becomes legal in US states, policies have expanded to include measures, such as regulating cost, strength, and types of cannabis products and where cannabis products can be sold.^1,2^ As part of this public health focus, some states where recreational cannabis is legal have enacted mandatory warning signs policies, requiring businesses to post point-of-sale signs that include a range of warnings about harms of using cannabis during pregnancy.^3,4,5^

However, it is not clear whether these warning signs are evidence-based. Research indicates that the proportion of pregnant people who use cannabis is increasing; whether this increase is related to legalization remains unclear.^6,7,8,9,10,11^ Research also indicates that use during pregnancy is associated with adverse child outcomes, including increased low birthweight and adverse child psychological outcomes.^12,13,14,15^ These increased risks have been found particularly for people who continue to use cannabis after they discover pregnancy and for people who use both tobacco and cannabis during their pregnancy.^12,14^ This body of evidence is likely sufficiently robust to justify public health strategies that disseminate evidence-based information about risks of using cannabis during pregnancy.

In contrast, there is very limited evidence assessing outcomes related to enacting warning signs policies. Research into mandatory warning sign policies for alcohol use during pregnancy may shed light on this question. While studies are mixed as to whether alcohol consumption during pregnancy is lower when alcohol warning signs are in effect,^16,17^ policies requiring alcohol warning signs are associated with increased adverse birth outcomes and decreased prenatal care utilization.^18,19^ Consistent with the alcohol warning signs and adverse birth outcomes findings, a 2022 analysis found that Washington state’s cannabis warning signs policy was associated with decreased birthweight and increased low birthweight.^20^

However, considerable questions remain as to why warning signs for substance use during pregnancy may have unintended adverse consequences. One possibility is that warning signs increase fears of punishment and thus influence pregnant people to avoid prenatal care. Another is that warning signs may lead people to believe their substance use has already irreversibly harmed their baby and thus it is too late to stop use. From the larger health communications literature, people who use cannabis could experience message fatigue^21^ and tune out or distrust information in messages. Research has not yet examined these mechanisms.

We examined possible mechanisms through which cannabis warning signs could impact birth outcomes, specifically whether they might relate to cannabis-related beliefs and use. In a sample of pregnant and recently pregnant people, we examined the hypotheses that exposure to warning signs is associated with greater beliefs that cannabis use during pregnancy (CUDP) is not safe, community stigma related to CUDP, message fatigue regarding CUDP, and support for punishing people who use cannabis during pregnancy. We explore whether warning signs were associated with CUDP and people’s perspectives on warning signs.

## Methods

This cross-sectional study was granted ethical approval from the University of California, San Francisco. Participants provided electronic informed consent. We followed the Strengthening the Reporting of Observational Studies in Epidemiology (STROBE) reporting guideline.

### Sample

Ipsos, a global market research firm, recruited participants from members of its web-based KnowledgePanel, a probability-based panel designed to be representative of the US based on US Census data. To reach a sufficient sample of pregnant and recently pregnant people, Ipsos also recruited participants from existing nonprobability panels.

Participants were eligible if they were noninstitutionalized English- and Spanish-speaking adults ages 18 to 49 years who were assigned female at birth, currently pregnant or recently pregnant (ie, within the past 2 years), and residing in a study state, including 36 US states and Washington, the District of Columbia (DC). Survey states included all states and Washington, DC, that had legalized recreational cannabis, including 15 states without warning signs policies (Alaska, California, Connecticut, DC, Maine, Massachusetts, Michigan, Montana, Nevada, New Jersey, New Mexico, New York, Rhode Island, Vermont, and Virginia) and 5 with warning signs policies (Arizona, Colorado, Illinois, Oregon, and Washington).^22,23^ The survey also included a purposively selected subset that had neither legal cannabis nor warning signs policies (Delaware, Hawaii, Idaho, Indiana, Iowa, Maryland, Minnesota, Missouri, New Hampshire, North Dakota, North Carolina, Ohio, Pennsylvania, South Dakota, Utah, Wisconsin, and Wyoming).^24^

Between May 23 and June 28, 2022, Ipsos invited eligible people to participate. Email reminders were sent up to 5 additional times to potential participants. For households using their own personal computers and internet service for survey participation, Ipsos enrolls panelists into a points program analogous to “frequent flyer” programs, in that respondents are credited with points in proportion to their regular survey participation. Panelists receive cash-equivalent checks approximately every 4 to 6 months in amounts reflecting their participation level, which commonly results in distributions of approximately $4 to $6 per month. For households provided with internet hardware and services, their panel loyalty incentive is the hardware and service.

The survey received 3571 valid survey responses. Responses from nonprobability participants based on questions designed to screen out fraudulent participants were excluded. These questions included items unlikely to be answered affirmatively by pregnant or recently pregnant people, such as whether a participant was living in a community for adults aged 55 years and older. Of KnowledgePanel members, 12 045 people were invited to participate; 6163 people (or 51.2%) completed the eligibility screener, of whom 747 people (12.1%) were eligible. For nonprobability panels, 8302 people completed the eligibility screener, of whom 2824 people (34.0%) were eligible. This higher proportion (34.0%) of eligible respondents likely reflects that 1 nonprobability panel prescreened participants so only currently and recently pregnant people were invited to participate.

### Sample Size Estimation

We estimated a sample size of 2400 in states with legal cannabis to detect a small to medium effect size between people in states with and without warning signs policies, assuming, based on preliminary data, that 25% of people would report using cannabis and recruitment at a 1:2 ratio in states with and without warning signs policies.

### Measures

Exposure to warning signs was measured in 2 ways, each dichotomous: living in a state with a warning signs policy (from the Alcohol Policy Information System^25^ and online searches for states enacting policies in 2021 or 2022)^26^ and reporting having seen a warning sign in the past 12 months. Outcomes included beliefs about CUDP and about warning signs: beliefs that use is not safe, perceived community stigma, support for punishment, warning signs perspectives, and message fatigue. To generate survey items, we reviewed peer-reviewed literature and obtained feedback from a community advisory board. This process resulted in 41 structured 5-point Likert-scaled items ranging from strongly disagree (−2) to strongly agree (2), with neither agree nor disagree in the middle and coded as 0. We assessed the Cronbach α for the 5 groups of items: beliefs that use is not safe, community stigma, support for punishment, warning signs perspectives, and message fatigue. Cronbach α for items related to beliefs and support for punishment were high (>.90). We removed 1 item to increase Cronbach α reliability for community stigma to .80. Warning signs perspectives (4 items) and message fatigue (4 items) had low Cronbach α (>.70); therefore, we examined individual items separately. For beliefs that use is not safe, community stigma, and support for punishment, higher scores indicate more negative beliefs about CUDP (ie, more beliefs that CUDP is not safe, more stigmatized in the community, and more support for punishment). The final scaled outcome variables were standardized and included: beliefs that CUDP is not safe (12 items; α = .92), perceived community stigma (6 items; α =0.80), and support for punishment (10 items; α = .93). Survey items are provided in eTable 1 in Supplement 1.

We also assessed CUDP as a dichotomous variable: used cannabis during their pregnancy vs not, based on reporting using cannabis daily or almost daily, weekly, monthly, or less than monthly during their pregnancy (if recently pregnant) or in the past 30 days (if currently pregnant) or if they responded yes that they “used cannabis at all during your pregnancy?” Individual-level control variables included pregnancy outcome, age, education, race and ethnicity, sexual or gender minority, gravidity, marital status, and whether in the past year they have seen public health messages or education campaigns about CUDP on billboards, brochures or posters, cannabis products or packages, social media, or websites. For race and ethnicity, people self-identified into categories defined by investigators, including Black, non-Hispanic; Hispanic; White, non-Hispanic; other, non-Hispanic (including American Indian or Alaska Native and Asian participants, as well as people who did not report race or reported another race in open-ended responses), and 2 or more races. Race and ethnicity were assessed to be able to account for variations in experiences and beliefs based on the social category of race and ethnicity, as a proxy for experiences of racism. The sexual or gender minority category included people who reported a sexual orientation other than straight or heterosexual (ie, gay or lesbian, bisexual, questioning, something else, and preferring not to answer) and people who reported a gender identity other than woman (ie, transgender man, man, nonbinary or gender nonconforming, something else, or preferring not to answer). Cannabis use was categorical (ie, no cannabis, reported not using cannabis 12 months before discovering pregnancy; cannabis before but not during, pregnancy, cannabis use 12 months before discovering, but not during, pregnancy; and cannabis during pregnancy). State-level control variables included unemployment rate, proportion of state residents below the poverty level, and 6 pregnancy-specific drug policies (ie, child abuse or neglect; child protective services reporting requirements; reporting requirements related to data; reporting requirements for assessments and treatment; priority treatment for pregnant people only and for pregnant people and women with children; and limits on criminal prosecution)^3^ in effect in 2020.^27^

### Statistical Analysis

For primary analyses, we restricted the sample to people living in states with legal recreational cannabis, as state governments typically consider warning signs once recreational cannabis is legal. We used multivariable linear and logistic regression, stratifying based on cannabis use, and controlling for individual-level covariates; warning signs policy models also controlled for state-level factors. Final models excluded education, as age and education were highly correlated. Warning signs policy models used generalized linear models with random effects for state and the appropriate functional form for the outcome (gaussian for beliefs scales; binomial for cannabis use). Warning signs models used linear and logistic regression, clustering SEs by state. We used casewise deletion for missing data, which was less than 0.1% in all cases except for the dichotomous cannabis use during pregnancy outcome, which was 0.6%. We conducted sensitivity analyses that included people from states without legal recreational cannabis in the no warning signs category and adding a state-level control for legal cannabis. Analyses of warning signs perspectives and message fatigue included χ^2^ tests.

All analyses were conducted in Stata statistical software version 17 (StataCorp), used a 2-sided statistical significance level of *P* < .05, and used survey weights, which weighted participants to be representative of the US population, based on the US census; therefore, numbers given are actual numbers and percentages are weighted. Data were analyzed from July 2022 to January 2023.

## Results

### Sample Description

A total of 3571 pregnant or recently pregnant people, including 2063 people in states with legal recreational cannabis (study states). Among participants in study states, the mean (SD) weighted age was 32 (6) years; 1421 participants (weighted, 71.0%) had a recent pregnancy that ended in a birth, 494 participants (weighted, 22.2%) were currently pregnant; and 148 participants (weighted, 6.8%) had a recent pregnancy that did not result in a birth (Table 1). There were 418 participants (weighted, 18.8%) who reported using cannabis before, but not during, their pregnancy, and 585 participants (weighted, 17.2%) reported using cannabis during their pregnancy. A total of 417 participants (weighted, 14.3%) reported having seen cannabis warning signs. Only age, education, and marital status varied across states by warning signs policies.

**Table 1.** Characteristics of Study Participants

Characteristic	Participants, No. (weighted %)			*P* value^a^
	Total	Living in a state without MWS-cannabis policy	Living in a state with MWS-cannabis policy	
Total	2063 (100)	1318 (75.3)	745 (24.7)	NA
Pregnancy status				
Currently pregnant	494 (22.2)	315 (22.6)	179 (20.9)	.78
Recent pregnancy that ended with birth	1421 (71.0)	907 (70.4)	514 (72.9)	
Recent pregnancy that did not end with birth	148 (6.8)	96 (7.0)	52 (6.2)	
Age, y				
18-20	85 (3.3)	43 (2.3)	42 (6.2)	<.001
21-24	279 (9.3)	167 (9.4)	112 (8.9)	
25-29	476 (22.7)	302 (22.7)	174 (22.5)	
30-34	578 (30.4)	371 (30.2)	207 (31.0)	
35-39	443 (22.4)	297 (22.6)	146 (21.9)	
40-49	202 (12.0)	138 (12.9)	64 (9.4)	
Education				
No high school	91 (4.4)	51 (4.1)	40 (5.1)	.03
High school graduate or GED	460 (20.7)	264 (18.9)	196 (26.0)	
Some college or associate degree	746 (26.7)	464 (26.4)	282 (27.7)	
Bachelor’s degree	488 (28.3)	340 (29.8)	148 (23.6)	
≥Master’s	278 (20.0)	199 (20.8)	79 (17.7)	
Race and ethnicity				
Black, non-Hispanic	187 (10.1)	132 (10.6)	55 (8.5)	.76
Hispanic	473 (23.5)	323 (24.0)	150 (22.2)	
White, non-Hispanic	1203 (53.0)	736 (51.7)	467 (56.7)	
Other, non-Hispanic^b^	112 (9.4)	72 (10.1)	40 (7.5)	
≥2 Races	88 (4.0)	55 (3.7)	33 (5.1)	
Sexual or gender minority^c^				
Yes	371 (14.3)	233 (13.5)	138 (16.9)	.15
No	1692 (85.7)	1085 (86.5)	607 (83.2)	
Previous pregnancies, No.				
0	513 (27.3)	343 (28.4)	170 (24.0)	.58
1	547 (26.9)	341 (26.6)	206 (27.6)	
2	420 (20.8)	266 (20.6)	154 (21.4)	
≥3	582 (25.0)	367 (24.4)	215 (27.0)	
Marital status				
Now married	1225 (70.0)	800 (70.5)	425 (68.5)	<.001
Widowed, divorced, or separated, not living with a partner	151 (5.0)	88 (4.7)	63 (5.8)	
Never married, not living with a partner	285 (9.8)	157 (8.6)	128 (13.6)	
Living with partner, not married	402 (15.2)	273 (16.2)	129 (12.2)	
Cannabis use				
No use before or during pregnancy	1059 (64.0)	706 (64.7)	353 (61.8)	.43
Use before, but not during pregnancy^d^	418 (18.8)	268 (18.8)	150 (19.0)	
Use during pregnancy^d^	585 (17.2)	343 (16.5)	242 (19.2)	
Seen MWS-cannabis signs				
Yes	417 (14.3)	233 (13.5)	184 (16.8)	.10
No	1645 (85.7)	1084 (86.5)	561 (83.2)	

Abbreviation: MWS-cannabis, mandatory warning signs about harms of cannabis use during pregnancy.

^a^ *P* values based on Wald tests from weighted bivariable logistic regressions that account for clustering by state.

^b^ Includes Asian, American Indian/Alaska Native, as well as people who did not report race or reported another race in open-ended responses.

^c^ People who did not affirmatively identify as cisgender and heterosexual were categorized as being a sexual or gender minority.

^d^ Pregnancy refers to the most recent pregnancy, ie, current or within the past 2 years.

### Cannabis Warning Signs Policies and Beliefs

In unadjusted models, warning signs policies were not associated with any beliefs outcome (eTable 2 in Supplement 1). In adjusted models, among people reporting CUDP, living in a state with a warning signs policy was associated with belief that CUDP was safe (β = −0.33 [95% CI, −0.60 to −0.07]) and with less support for punishment (β = −0.40 [95% CI, −0.73 to −0.07]), but not with community stigma. Among people reporting no cannabis use, living in a state with a warning signs policy was associated with belief that CUDP is not safe (β = 0.34 [95% CI, 0.17 to 0.51]), more perceived stigma (β = 0.35 [95% CI, 0.07 to 0.63]), and more support for punishment (β = 0.35 [95% CI, 0.24 to 0.47]) (Table 2). Among people reporting cannabis use before, but not during, pregnancy, there were no statistically significant associations between warning signs policies and any beliefs outcome.

**Table 2.** Association of MWS-Cannabis Exposure With Beliefs About Cannabis Use During Pregnancy Among Individuals Living in States With Legal Recreational Cannabis

Cannabis use subgroup	β (95% CI) (n = 2063)^a^		
	Belief that use during pregnancy is not safe	Perceived community stigma	Support for punishment
**MWS-cannabis policy** ^b^			
Use during pregnancy	−0.33 (−0.60 to −0.07)	−0.31 (−0.75 to 0.13)	−0.40 (−0.73 to −0.07)
Use before, but not during, pregnancy	−0.06 (−0.37 to 0.24)	−0.22 (−0.67 to 0.23)	−0.02 (−0.26 to 0.22)
No use before or during pregnancy	0.34 (0.17 to 0.51)	0.35 (0.07 to 0.63)	0.35 (0.24 to 0.47)
**MWS-cannabis signs** ^c^			
Use during pregnancy	0.06 (−0.14 to 0.27)	−0.01 (−0.30 to 0.28)	0.16 (−0.04 to 0.35)
Use before, but not during, pregnancy	−0.31 (−0.57 to −0.06)	0.12 (−0.25 to 0.5)	−0.31 (−0.62 to 0.01)
No use before or during pregnancy	−0.28 (−0.61 to 0.04)	−0.20 (−0.49 to 0.09)	0.02 (−0.24 to 0.27)

Abbreviation: MWS-cannabis, mandatory warning signs about cannabis use during pregnancy.

^a^ Range, −2 to 2. Higher scores indicate more negative beliefs about cannabis use during pregnancy (ie, more beliefs that use is not safe, that use is more stigmatized, and more support for punishment).

^b^ Controls for state-level pregnancy-specific drug policies, unemployment, and poverty; and individual-level age, pregnancy outcome for most recent pregnancy, race and ethnicity, sexual or gender minority, gravidity, and marital status.

^c^ Controls for individual-level reporting having seen cannabis and pregnancy education or messages on billboards, brochures, products, social media, websites, and other locations, as well as age, pregnancy outcome for most recent pregnancy, race and ethnicity, sexual or gender minority, gravidity, and marital status.

### Cannabis Warning Signs and Beliefs

In unadjusted models, only 1 association, between having seen warning signs and support for punishment among people reporting CUDP, was statistically significant (eTable 2 in Supplement 1). In adjusted models, among people reporting using cannabis before, but not during, pregnancy, having seen warning signs was associated with belief that CUDP during pregnancy was safe (β = −0.31 [95% CI, −0.57 to −0.06]) (Table 2). Among people reporting CUDP and among people reporting no use, having seen warning signs was not associated with any beliefs outcome.

### Cannabis Warning Signs Policies, Seeing Warning Signs, and Cannabis Use

In unadjusted (eTable 3 in the Supplement) and adjusted (Table 3) models, living in a state with a warning signs policy was not associated with CUDP (adjusted odds ratio [aOR], 1.11 [95% CI, 0.22 to 5.67]) overall or when restricted to people who used cannabis before or during pregnancy (aOR, 1.49 [95% CI, 0.47 to 4.69]). In unadjusted and adjusted models, having seen warning signs was associated with CUDP overall (aOR, 1.53 [95% CI, 1.02 to 2.27]) but not when restricted to people reporting use before or during pregnancy (aOR, 1.25 [95% CI, 0.78 to 2.00]).

**Table 3.** Association of MWS-Cannabis Exposure and Cannabis Use During Pregnancy in the Full Sample and Participants Who Reported Using Before or During Pregnancy for People Living in States With Legal Recreational Cannabis

Sample	Use during pregnancy, aOR (95% CI)
MWS-cannabis policy^a^	
Full sample (n = 2048)	1.11 (0.22-5.67)
Use before or during pregnancy (n = 1004)	1.49 (0.47-4.69)
MWS-cannabis signs^b^	
Full sample (n = 2048)	1.53 (1.02-2.27)
Use before or during pregnancy (n = 1004)	1.25 (0.78-2.00)

Abbreviation: MWS-cannabis, mandatory warning signs about the harms of cannabis use during pregnancy.

^a^ Controls for state-level pregnancy-specific drug policies, unemployment, and poverty; and individual-level age, pregnancy outcome for most recent pregnancy, race and ethnicity, sexual or gender minority, gravidity, and marital status.

^b^ Controls for individual-level reporting having seen cannabis and pregnancy education or messages on billboards, brochures, products, social media, websites, other locations as well as age, pregnancy outcome for most recent pregnancy, race and ethnicity, sexual or gender minority, gravidity, and marital status.

### Sensitivity Analyses

Sensitivity analyses were substantively similar with 2 exceptions: warning signs policy and outcomes among people reporting no use, in which no association remained statistically significant; and having seen warning signs and support for punishment among people reporting use before, but not during pregnancy, in which having seen warning signs was associated with less support for punishment (β = −0.32 [95% CI, −0.59 to −0.06]) (eTable 4 and eTable 5 in Supplement 1).

### Warning Signs Perspectives and Message Fatigue

**Figure 1.**
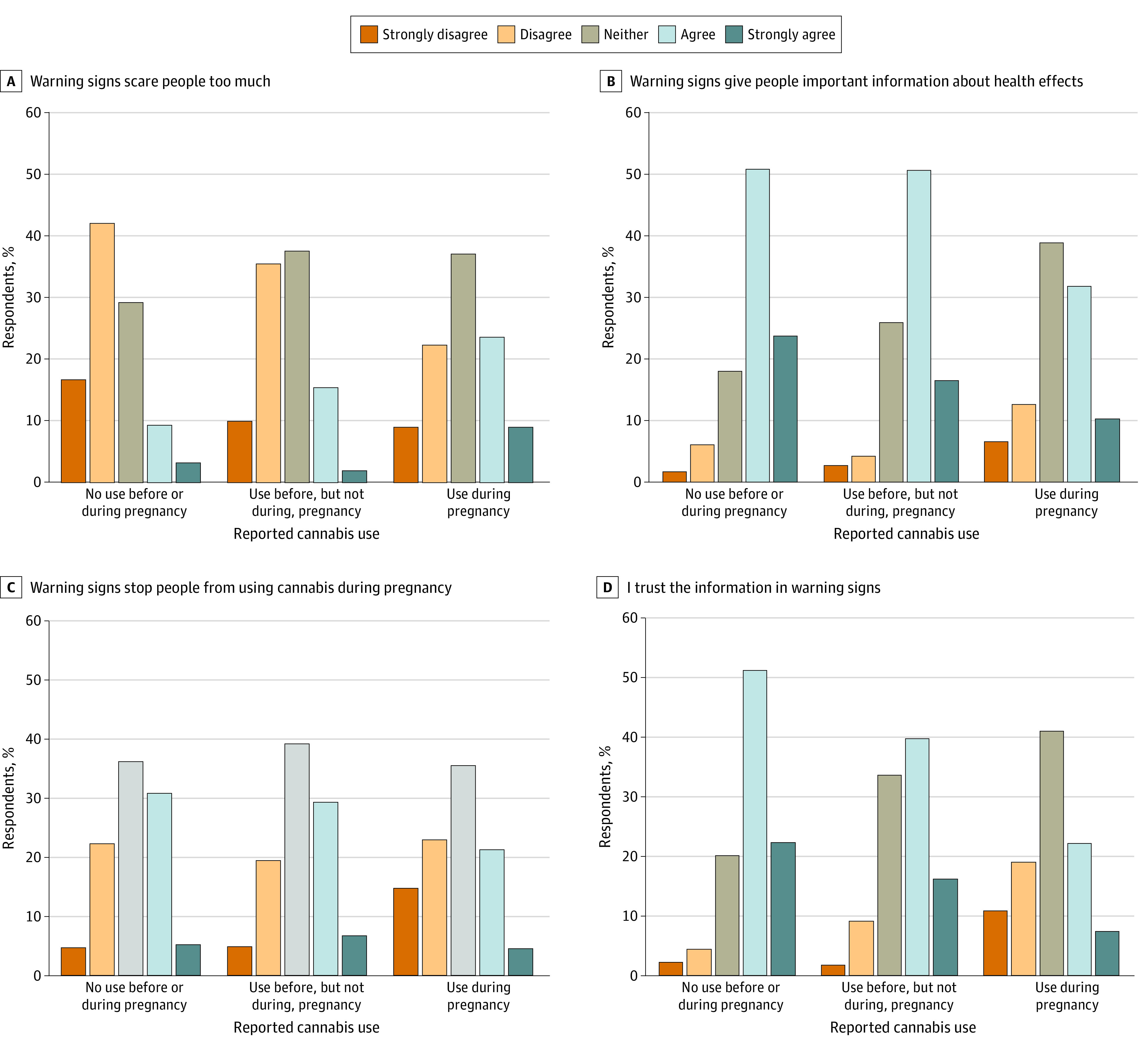
Perspectives on Warning Signs by Cannabis Use Category

**Figure 2.**
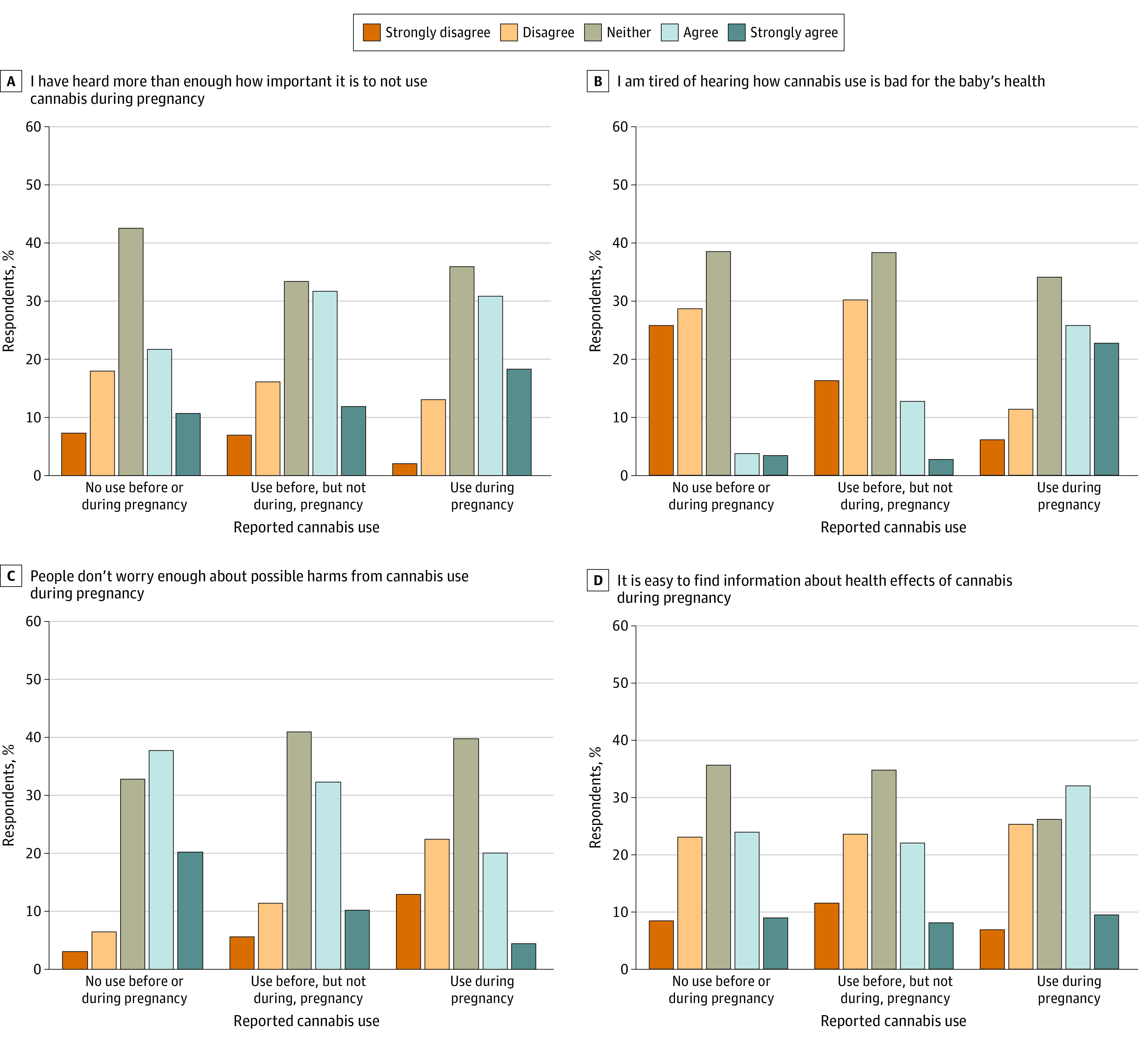
Message Fatigue by Cannabis Use Category

Of 585 people reporting CUDP, 226 participants (weighted, 32.4%) agreed that warning signs scare people too much, while 268 participants (weighted, 42.1%) agreed that warning signs give people important information, 157 participants (weighted, 25.9%) believed signs stop people from using cannabis during pregnancy, and 171 participants (weighted, 29.3%) trusted information in signs (Figure 1). Regarding message fatigue, of people reporting CUDP, 302 participants (weighted, 48.4%) agreed they were tired of hearing how cannabis is bad for their baby’s health, 266 participants (weighted, 49.1%) agreed they have heard more than enough about how important it is to not use during pregnancy, 250 participants (weighted, 41.5%) agreed that it is easy to find information about health effects of cannabis during pregnancy; while only 140 participants (weighted, 24.4%) agreed that people do not worry enough about possible harms from CUDP (Figure 2). Cannabis use was associated with all warning signs perspectives and message fatigue items except ease of finding trustworthy information. People reporting CUDP endorsed more negative perspectives on warning signs and more message fatigue than people reporting cannabis use before, but not during, pregnancy (although the difference for having heard more than enough about how important it is to not use during pregnancy was not statistically significant); both cannabis use groups endorsed more negative warning signs perspectives and more message fatigue than people who did not report cannabis use.

## Discussion

This cross-sectional study found that among people who used cannabis during pregnancy, living in a state with a warning signs policy was associated with believing that using cannabis during pregnancy was safe and should not be punished. In contrast, among people who did not use cannabis, living in a state with a warning signs policy was associated with believing cannabis use during pregnancy was not safe, that people who used cannabis during pregnancy should be punished, and with greater community stigma regarding pregnant people’s cannabis use. Among people who used cannabis during pregnancy, we observed message fatigue related to messages about possible harms of using cannabis during pregnancy and mistrust of information in warning signs.

We also found no evidence that exposure to warning signs was associated with decreased cannabis use during pregnancy. The finding that having seen warning signs was associated with increased odds of cannabis use during pregnancy among the overall sample likely reflects that only people who use cannabis likely see warning signs, as signs are in specialized dispensaries, visited by people who use cannabis. The possibility that exposure to warning signs could affect quantity or frequency of use during pregnancy is something to explore in future research.

It is worth noting that, among people using cannabis, exposure to warning signs was associated with believing cannabis use during pregnancy was safe and should not be punished, which is opposite of our hypotheses. Combined with the finding that people who used cannabis during pregnancy expressed message fatigue and mistrust of information in warning signs, it seems possible that cannabis warning signs may (inadvertently) communicate that cannabis use during pregnancy is safe, people do not trust information in warning signs, or because of message fatigue, people are not in a position to take in the information provided by warning signs.

We had also hypothesized that warning signs exposure would be associated with more community stigma and stronger support for punishment. While we did not find these associations among people who used cannabis, we did find these associations among people who did not use cannabis, although this finding was sensitive to exclusion of people living in states without legal recreational cannabis. This finding suggests that warning signs might relate to broader community attitudes in legal recreational cannabis states, such as interactions pregnant people who use cannabis may have in their community, including with health care practitioners. Research suggests that legalizing cannabis does not make pregnant people comfortable talking with health care practitioners about cannabis^28,29^; future research should explore ways warning signs may influence interactions between pregnant people and health care practitioners.

Regarding sample size, the proportion reporting cannabis use was greater than original estimates. Thus, although the actual sample was smaller than anticipated, sufficient numbers reported cannabis use to detect small to medium effect sizes.

### Limitations

This study has a few limitations. First, the inclusion of both probability and nonprobability panel members limits the ability to estimate participation rates and thus assess generalizability of our sample. However, for answering our main study question, participants living in states with vs without warning signs policies were demographically similar. Nonprobability panel participants with more experience with the topic may have been more likely to participate; thus, the proportion reporting cannabis use should not be used as a prevalence estimate. It is also worth noting that we assessed use at any point in pregnancy, whereas other national surveys ask pregnant people about past-month use.^30^ Second, due to the cross-sectional design, we cannot know the direction of associations between having seen warning signs and outcomes. Only people who use cannabis are likely to be exposed to the warning signs, and people who use more frequently may be even more likely to be exposed, given that warning signs are posted in cannabis dispensaries, locations visited by people who use cannabis. Our use of living in a state with a warning signs policy as another way of measuring exposure helps mitigate this concern. Third, policy data are not aligned with the precise timing of when people were pregnant. Fourth, 3 of the states with warning signs policies have had legal recreational cannabis for relatively longer time periods,^25^ which could also explain findings.

## Conclusions

This cross-sectional study found that among people who used cannabis, living in a state with warning signs policies was not associated with reduced cannabis use during pregnancy or with believing use was less safe. However, living in a state with a warning signs policy may be associated with greater stigma and support for punishment among the broader community.

## Notes

Supplement 1. **eTable 1.** Factor Analysis of Cannabis Pregnancy Beliefs, Stigma, and Attitudes Towards Punishment (n = 3571) **eTable 2.** Association of Mandatory Warning Signs Exposure With Beliefs About Cannabis Use During Pregnancy, Unadjusted (n = 2063) **eTable 3.** Association of Mandatory Warning Signs Exposure With During Pregnancy, Unadjusted (n = 2048 Full Sample and n = 1004 Among Those Using Before or During Pregnancy) **eTable 4.** Association of Mandatory Warning Signs Exposure With Beliefs About Cannabis Use During Pregnancy (n = 3571) **eTable 5.** Association of Mandatory Warning Signs Exposure With Cannabis Use During Pregnancy (n = 3549 Full Sample and n = 1631 Among Those Using Before or During Pregnancy)

Supplement 2.
Data Sharing Statement
