# Supplementary material for: Association of Mandatory Warning Signs for Cannabis Use During Pregnancy With Cannabis Use Beliefs and Behaviors
Source: JAMA Netw Open. 2023 Jun 14;6(6):e2317138. doi: 10.1001/jamanetworkopen.2023.17138 (PMC10267765; doi:10.1001/jamanetworkopen.2023.17138)
Supplement: Supplement 1. — eTable 1. Factor Analysis of Cannabis Pregnancy Beliefs, Stigma, and Attitudes Towards Punishment (n = 3571) eTable 2. Association of Mandatory Warning Signs Exposure With Beliefs About Cannabis Use During Pregnancy, Unadjusted (n = 2063) eTable 3. Association of Mandatory Warning Signs Exposure With During Pregnancy, Unadjusted (n = 2048 Full Sample and n = 1004 Among Those Using Before or During Pregnancy) eTable 4. Association of Mandatory Warning Signs Exposure With Beliefs About Cannabis Use During Pregnancy (n = 3571) eTable 5. Association of Mandatory Warning Signs Exposure With Cannabis Use During Pregnancy (n = 3549 Full Sample and n = 1631 Among Those Using Before or During Pregnancy) [file jamanetwopen-e2317138-s001.pdf]

Supplemental Online Content

Roberts SCM, Zaugg C, Biggs MA. Association of mandatory warning signs for cannabis use during pregnancy with cannabis use beliefs and behaviors. *JAMA Netw Open*. 2023;6(6):e2317138. doi:10.1001/jamanetworkopen.2023.17138

eTable 1. Factor Analysis of Cannabis Pregnancy Beliefs, Stigma, and Attitudes Towards Punishment (n = 3571)

eTable 2. Association of Mandatory Warning Signs Exposure With Beliefs About Cannabis Use During Pregnancy, Unadjusted (n = 2063)

eTable 3. Association of Mandatory Warning Signs Exposure With During Pregnancy, Unadjusted (n = 2048 Full Sample and n = 1004 Among Those Using Before or During Pregnancy)

eTable 4. Association of Mandatory Warning Signs Exposure With Beliefs About Cannabis Use During Pregnancy (n = 3571)

eTable 5. Association of Mandatory Warning Signs Exposure With Cannabis Use During Pregnancy (n = 3549 Full Sample and n = 1631 Among Those Using Before or During Pregnancy)

This supplemental material has been provided by the authors to give readers additional information about their work.

© 2023 Roberts SCM et al. *JAMA Network Open.*

**eTable 1.** Factor Analysis of Cannabis Pregnancy Beliefs, Stigma, and Attitudes Towards Punishment (n = 3571)	
Item	
During pregnancy, cannabis is safer than alcohol	
People who use cannabis during pregnancy can have a healthy baby	
During pregnancy, cannabis is safer than tobacco	
People who use cannabis during pregnancy can be great parents	
When people use cannabis during pregnancy, their babies are usually just fine	
During pregnancy, cannabis is safer than other medications to treat nausea or anxiety	
Because cannabis comes from a plant, it is safe to use during pregnancy	
Using cannabis during pregnancy is safe for the baby	
Using cannabis during pregnancy can cause birth defects	
Cutting back on cannabis use during pregnancy is good for the baby’s health	
Stopping cannabis use any time during pregnancy is good for the baby’s health	
Using cannabis during pregnancy can cause babies to be born too small	
**Beliefs that cannabis use during pregnancy is unsafe**	**Alpha=0.92**
Pregnant people in my community do not want other people to know that they use cannabis	
Pregnant people in my community hide their cannabis use from their prenatal care providers	
Pregnant people in in my community who use cannabis worry that they will be judged	
Pregnant people in my community who use cannabis worry that their prenatal care providers will report them to Child Protective Services (CPS)	
Pregnant people in my community are comfortable talking with their prenatal care providers about cannabis	
Pregnant people in my community are comfortable with other people knowing that they are using cannabis	
**Presence of community stigma related to pregnant people’s cannabis use**	**Alpha=0.80**
Child Protective Services (CPS) should leave pregnant people who use cannabis alone	
Pregnant people who use cannabis should be reported to Child Protective Services (CPS)	
The health department should leave pregnant people who use cannabis alone	
Police officers should leave pregnant people who use cannabis alone	
Using cannabis during pregnancy is child abuse	
Cannabis use should be illegal for pregnant people	
Doctors and nurses should not test pregnant people’s urine for cannabis	
Doctors and nurses should get consent from the pregnant person before testing their urine for cannabis	
Pregnant people should be able to talk with their doctor about their cannabis use without worrying about getting in trouble	
Cannabis use should be illegal for everyone	
**Attitudes supporting punishment of pregnant people’s cannabis use**	**Alpha=0.93**

© 2023 Roberts SCM et al. *JAMA Network Open.*

**eTable 2.** Association of Mandatory Warning Signs Exposure With Beliefs About Cannabis Use During Pregnancy,[a] Unadjusted (n = 2063)										
		Belief that use during pregnancy is not safe (range -2, 2)			Perceived community stigma (range -2, 2)			Support for punishment (range -2, 2)		
Cannabis use subgroup	*Exposure*	β	95% CI		β	95% CI		β	95% CI	
**Use during pregnancy**	MWS-cannabis policy	-0.12	-0.42	0.18	0.13	-0.28	0.54	-0.19	-0.54	0.16
**Use before, but not during, pregnancy**	MWS-cannabis policy	-0.10	-0.42	0.21	-0.02	-0.16	0.13	-0.19	-0.39	0.01
**No use before or during pregnancy**	MWS-cannabis policy	0.04	-0.11	0.19	-0.01	-0.22	0.21	0.04	-0.12	0.22
**Use during pregnancy**	MWS-cannabis signs	0.15	-0.09	0.39	-0.11	-0.40	0.18	0.31	0.06	0.55
**Use before, but not during, pregnancy**	MWS-cannabis signs	-0.17	-0.47	0.13	0.07	-0.31	0.45	-0.12	-0.33	0.10
**No use before or during pregnancy**	MWS-cannabis signs	-0.26	-0.53	0.01	0.03	-0.24	0.30	-0.06	-0.29	0.17

[a] Sample includes people living in states with legalized recreational cannabis
Higher scores indicate more negative beliefs about cannabis use during pregnancy (i.e. more beliefs that use is not safe, that use is more stigmatized, and more support for punishment)
abbreviations: MWS-cannabis, mandatory warning signs for cannabis use during pregnancy

© 2023 Roberts SCM et al. *JAMA Network Open.*

eTable 3. Association of Mandatory Warning Signs Exposure With During Pregnancy,[a] Unadjusted (n = 2048 Full Sample and n = 1004 Among Those Using Before or During Pregnancy)

		Use during pregnancy		
Sample	*Exposure*	aOR	95% CI	
**Full sample**	MWS-cannabis policy	1.29	0.82	2.04
**Use before or during pregnancy**	MWS-cannabis policy	1.20	0.71	2.05
**Full sample**	MWS-cannabis signs	3.55	2.52	5.02
**Use before or during pregnancy**	MWS-cannabis signs	1.34	0.95	1.91

[a] Sample includes people living in states with legalized recreational cannabis

abbreviations: MWS-cannabis, mandatory warning signs for cannabis use during pregnancy

© 2023 Roberts SCM et al. *JAMA Network Open.*

eTable 4. Association of Mandatory Warning Signs Exposure With Beliefs About Cannabis Use During Pregnancy[a] (n = 3571)

		Belief that use during pregnancy is not safe (range -2, 2)			Perceived community stigma (range -2, 2)			Support for punishment (range -2, 2)		
Cannabis use subgroup	*Exposure*	β	95% CI		β	95% CI		β	95% CI	
Use during pregnancy	MWS-cannabis policy[b]	-0.22	-0.45	0.01	0.13	-0.32	0.57	-0.32	-0.57	-0.07
Use before, but not during, pregnancy	MWS-cannabis policy[b]	0.04	-0.14	0.22	-0.06	-0.36	0.24	-0.12	-0.32	0.09
No use before or during pregnancy	MWS-cannabis policy[b]	0.01	-0.19	0.22	0.20	-0.03	0.43	0.06	-0.12	0.24
Use during pregnancy	MWS-cannabis signs[c]	-0.00	-0.18	0.18	0.00	-0.20	0.21	0.08	-0.12	0.28
Use before, but not during, pregnancy	MWS-cannabis signs[c]	-0.31	-0.50	-0.12	0.12	-0.18	0.42	-0.32	-0.59	-0.06
No use before or during pregnancy	MWS-cannabis signs[c]	-0.15	-0.40	0.09	-0.21	-0.46	0.05	0.02	-0.20	0.24

[a] Sample includes people living in all included states

[b] Controls for state-level: pregnancy-specific drug policies, unemployment, poverty; and individual-level: age, pregnancy outcome for most recent pregnancy, race/ethnicity, sexual/gender minority, gravidity, and marital status

[c] Controls for individual-level: reporting having seen cannabis and pregnancy education/messages on billboards, brochures, products, social media, websites, other locations as well as age, pregnancy outcome for most recent pregnancy, race/ethnicity, sexual/gender minority, gravidity, and marital status

Higher scores indicate more negative beliefs about cannabis use during pregnancy (i.e. more beliefs that use is not safe, that use is more stigmatized, and more support for punishment)

© 2023 Roberts SCM et al. *JAMA Network Open.*

eTable 5. Association of Mandatory Warning Signs Exposure With Cannabis Use During Pregnancy[a] (n = 3549 Full Sample and n = 1631 Among Those Using Before or During Pregnancy)

		Use during pregnancy		
Sample	*Exposure*	aOR	95% CI	
Full sample	MWS-cannabis policy[b]	0.92	0.38	2.25
Use before or during pregnancy	MWS-cannabis policy[b]	0.85	0.38	1.89
Full sample	MWS-cannabis signs[c]	1.39	1.00	1.93
Use before or during pregnancy	MWS-cannabis signs[c]	0.95	0.58	1.54

[a] Sample includes people living in all included states

[b] Controls for state-level: pregnancy-specific drug policies, unemployment, poverty; and individual-level: age, pregnancy outcome for most recent pregnancy, race/ethnicity, sexual/gender minority, gravidity, and marital status

[c] Controls for individual-level: reporting having seen cannabis and pregnancy education/messages on billboards, brochures, products, social media, websites, other locations as well as age, pregnancy outcome for most recent pregnancy, race/ethnicity, sexual/gender minority, gravidity, and marital status

© 2023 Roberts SCM et al. *JAMA Network Open.*
