# Supplementary material for: Association of Mandatory Warning Signs for Cannabis Use During Pregnancy With Cannabis Use Beliefs and Behaviors
Source: JAMA Netw Open. 2023 Jun 14;6(6):e2317138. doi: 10.1001/jamanetworkopen.2023.17138 (PMC10267765; doi:10.1001/jamanetworkopen.2023.17138)
Supplement: Supplement 2. — Data Sharing Statement [file jamanetwopen-e2317138-s002.pdf]

Data Sharing Statement

Roberts. Association of Mandatory Warning Signs for Cannabis Use During Pregnancy With Cannabis Use Beliefs and Behaviors. *JAMA Netw Open*. Published June 14, 2023.
doi:10.1001/jamanetworkopen.2023.17138

Data
Data available: No

Additional Information
Explanation for why data not available: De-identified data about cannabis related beliefs are available to qualified researchers interested in conducting additional analyses. Data about cannabis use behavior is not available to other researchers. This is because there is significant stigma and punishment experienced by pregnant people who use cannabis, and a breach in confidentiality for study participants could have severe consequences.
